# Metal free synthesis of ethylene and propylene carbonate from alkylene halohydrin and CO_2_ at room temperature†

**DOI:** 10.1039/c9ra06765e

**Published:** 2019-10-22

**Authors:** Santosh Govind Khokarale, Jyri-Pekka Mikkola

**Affiliations:** Technical Chemistry, Department of Chemistry, Chemical-Biological Centre, Umeå University SE-90187 Umeå Sweden santosh.khokarale@umu.se; Industrial Chemistry & Reaction Engineering, Department of Chemical Engineering, Johan Gadolin Process Chemistry Centre, Åbo Akademi University FI-20500 Åbo-Turku Finland

## Abstract

Herein we describe a metal free and one-pot pathway for the synthesis of industrially important cyclic carbonates such as ethylene carbonate (EC) and propylene carbonates (PC) from molecular CO_2_ under mild reaction conditions. In the actual synthesis, the alkylene halohydrins such as alkylene chloro- or bromo or iodohydrin and organic superbase, 1,8-diazabicyclo-[5.4.0]-undec-7-ene (DBU) reacted equivalently with CO_2_ at room temperature. The syntheses of cyclic carbonates were performed in DMSO as a solvent. Both 1,2 and 1,3 halohydrin precursors were converted into cyclic carbonates except 2-bromo- and iodoethanol, which were reacted equivalently with DBU through *n*-alkylation and formed corresponding *n*-alkylated DBU salts instead of forming cyclic carbonates. NMR analysis was used to identify the reaction components in the reaction mixture whereas this technique was also helpful in terms of understanding the reaction mechanism of cyclic carbonate formation. The mechanistic study based on the NMR analysis studies confirmed that prior to the formation of cyclic carbonate, a switchable ionic liquid (SIL) formed *in situ* from alkylene chlorohydrin, DBU and CO_2_. As a representative study, the synthesis of cyclic carbonates from 1,2 chlorohydrins was demonstrated where the synthesis was carried out using chlorohydrin as a solvent as well as a reagent. In this case, alkylene chlorohydrin as a solvent not only replaced DMSO in the synthesis but also facilitated an efficient separation of the reaction components from the reaction mixture. The EC or PC, [DBUH][Cl] as well as an excess of the alkylene chlorhydrin were separated from each other following solvent extraction and distillation approaches. In this process, with the applied reaction conditions, >90% yields of EC and PC were achieved. Meanwhile, DBU was recovered from *in situ* formed [DBUH][Cl] by using NaCl saturated alkaline solution. Most importantly here, we developed a metal free, industrially feasible CO_2_ capture and utilization approach to obtain EC and PC under mild reaction conditions.

## Abstract 2

Metal free, one-pot and room temperature syntheses of the industrially important cyclic carbonates such as ethylene and propylene carbonate were performed from alkylene halohydrins and CO_2_.

## Graphical abstract

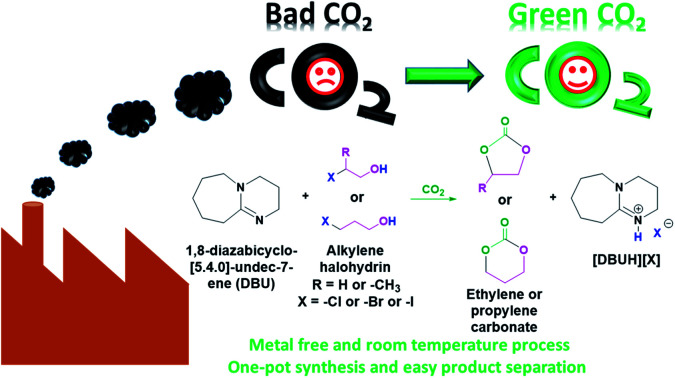

## 1. Introduction

**Fig. 1:**
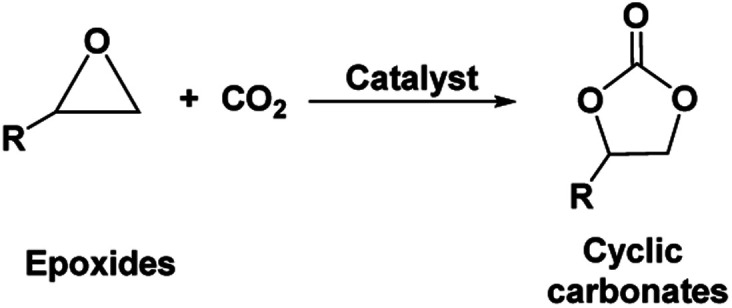
Synthesis of cyclic carbonates from epoxides and CO_2_.

Cyclic carbonates, especially ethylene and propylene carbonate are industrially important chemicals considering their widespread application.^1^ Being non-toxic and bio-degradable and having high boiling points and aprotic polar nature, these cyclic carbonates are preferentially replacing other harmful organic solvents such as dimethylformamide (DMF), hexamethylphosphoramide (HMPA), *N*-methyl-2-pyrrolidone (NMP), dimethylacetamide and acetonitrile (ACN).^1–3^ Cyclic carbonates have also been successfully implemented as intermediates upon synthesis of fine chemicals such as dialkyl carbonates, glycols, carbamates *etc.* Further, they find use as electrolytes for batteries, precursors for polymeric materials and as fuel additives.^4^ The traditional industrial production of cyclic carbonates follows the phosgene route. However, due to the toxic and corrosive nature of phosgene as well as production of large amounts of chlorinated salts and solvents, this process is not found to be sustainable in terms of environmental impact and work safety. As an alternative approach, the synthesis of cyclic carbonates from epoxides and carbon dioxide (CO_2_) was invented as an elegant, non-toxic and atom-efficient carbon capture and utilization (CCU) pathway (Fig. 1).^5^ This pathway introduced an opportunity to use CO_2_ as an inexpensive, nontoxic, and renewable carbon C1 building block for the synthesis of valuable chemicals. However, considering its more oxidized state and hence chemically inert nature, the CO_2_ molecule usually gives rise to low reactivity. Hence, for the chemical activation of CO_2_ molecule and its subsequent interaction with epoxides, several homogeneous and heterogeneous catalytic systems including metal based catalysts, organocatalysts, ionic liquids *etc.* were developed to synthesize cyclic carbonates.^4–8^

Apart from the catalytic systems, various types of epoxide substrates were also successfully used. These include both petroleum (for *e.g.* ethylene oxide, propylene oxide and cyclohexene oxide) as well as bio-based epoxides (for *e.g.* epichlorohydrine, limonene oxide and vinylcyclohexene oxide).^6,9–11^ Even though the synthesis of cyclic carbonates from various epoxides is industrially applied process but the synthesis of epoxides accompanied with the use of toxic or costly reagents as well as involves a tedious workup for separation.

Alternative to epoxide as a reagent, the synthesis of cyclic carbonates was also carried out using alkylene halohydrins as a one of the reagent with CO_2_. The various alkali metal carbonates such as K_2_CO_3_, Na_2_CO_3_, Cs_2_CO_3_*etc.* were used as an catalysts for the synthesis of the cyclic carbonates from various alkylene halohydrins. In this case the cyclic carbonates were obtained with high yield (>90%) under mild reaction conditions such as room temperature and atmospheric pressure.^12–15^ The metal free reaction approach for the synthesis of cyclic carbonates from alkylene halohydrins was also used where amine such as triethylamine was used as a both activating agent and solvent.^16^ Even though these reaction approaches emerged as an alternative to the epoxide based processes but it is also accompanied with various challenges. In case of metal carbonate based processes involves the use of organic solvents (*e.g.* dimethylformamide, DMF) and additives (*e.g.* amines) as well as the generation of equivalent amount of waste such as metal bicarbonates and metal halides. In addition, it was also observed that the desired cyclic carbonates were formed with slow reaction kinetics under applied reaction conditions. Further with triethylamine-involved processes, even though 90% yield of cyclic carbonates was obtained and recovery of amine was achieved, but the process was carried out at high temperature and pressure. Hence, the synthesis of cyclic carbonates from alkylene halohydrins need few improvements where combined reaction approach involving mild reaction conditions along with no metal waste generation can be more preferred.

**Scheme 1:**
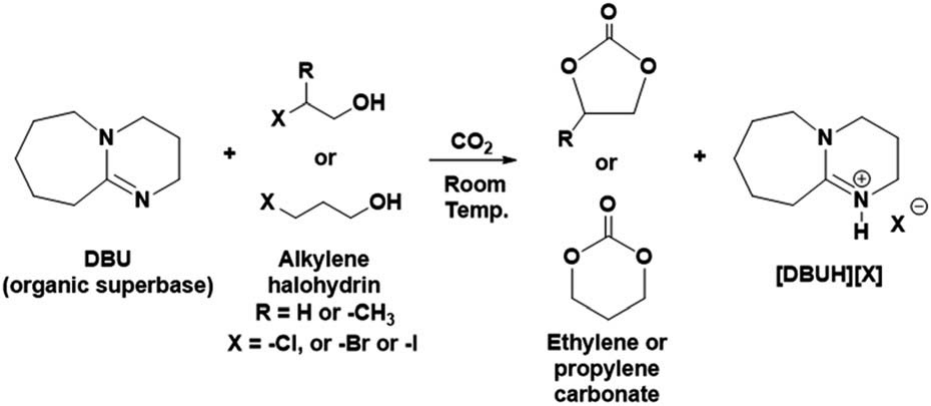
Synthesis of ethylene or propylene carbonate from alkylene halohydrins.

Herein, we are introducing one-pot and metal free synthesis of ethylene carbonate (EC) and propylene carbonate (PC) from molecular CO_2_ at room temperature. In this route, the equivalent mixture of various alkylene halohydrin such as 2-chloroethanol or 2-bromoethanol or 2-iodoethanol or 1-chloro-2-propanol or 3-chloro-1-propanol or 3-bromo-1-propanol and organic superbase DBU react with molecular CO_2_ to form EC or PC (reaction Scheme 1).

The process described in this report for the synthesis of EC and PC uses equivalent amounts of DBU, alkylene halohydrin and CO_2_ where synthesis was carried out at room temperature and atmospheric pressure. In addition, as shown in the reaction Scheme 1, the organic superbase DBU was recovered in the form of its halide salt. Further, the cyclic carbonates and salt of DBU were separated in the pure form with mild solvent extraction and vacuum distillation approach. The DBU was also recovered from its halide salt following alkaline solvent's treatment.

**Fig. 2:**
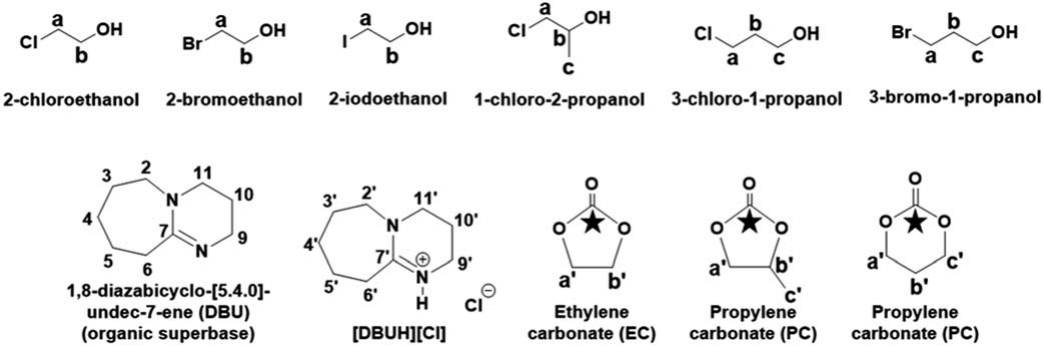
Chemical species used and formed in the reaction mixture in cyclic carbonate synthesis.

The synthesis of EC and PC was initially carried out in DMSO (as a solvent) while in order avoid to use of DMSO, the same process was further developed to yield a DMSO free solvent system where alkylene halohydrins themselves were used as solvents. The progress of the reaction as well as recovery of EC or PC and DBU were monitored by the means of NMR spectroscopic techniques. The numbering and labelling for the chemical species used and formed in this cyclic carbonate synthesis are shown in Fig. 2.

## 2. Experimental

### 2.1 Chemicals and methods

#### 2.1.1 Chemicals

1,8-Diazabicyclo-[5.4.0]-undec-7-ene (DBU, ≥99.0%, GC analysis), 2-chloroethanol (≥99.0%), 2-bromoethanol (95%), 2-iodoethanol (98%), propylene chlorohydrin (70 wt% of 1-chloro-2-propanol and 30% of 2-chloro-1-propanol), 3-chloro-1-propanol (98%), 3-bromo-1-propanol (98%) and D_2_O (99.9 atom % D), d^6^-DMSO (anhydrous, 99.9 atom% D) were purchased from Sigma Aldrich while ethyl acetate (≥99.0%), ethanol (≥99.0%), and dimethyl sulfoxide (≥99.0%) were purchased from VWR chemicals and used further without purification. CO_2_ and ^13^C enriched CO_2_ gas bottles (>99.99%) were obtained from AGA AB (Linde Group) and used without further purification.

#### 2.1.2 NMR analysis

The composition of the reaction mixtures as well as purity of the recovered ethylene carbonate, propylene carbonate and DBU were confirmed by the means of NMR analysis using the Brucker's Avance 400 MHz instrument. In these analyses, as per requirement, either glass capillary filled with D_2_O or DMSO-d^6^ were used as internal reference. The obtained data was further processed with TopSpin 3.2 software.

### 2.2. Synthesis and separation of MP and MMA and recovery of DBU

#### 2.2.1 Synthesis of ethylene or propylene carbonate with and without dimethyl sulfoxide (DMSO) as a solvent

The ethylene carbonate (EC) or propylene carbonates (PC) were prepared from various halohydrins, DBU and CO_2_ with or without DMSO solvent. In this synthesis, CO_2_ gas was bubbled (50 ml min^−1^) for 20 min under stirring in the DMSO solution containing equimolar amounts of DBU and halohydrin at room temperature. In case of DMSO free experiments, the CO_2_ gas was bubbled (50 ml min^−1^) for 20 min in chlorohydrin solution of DBU (50 vol% of DBU in chlorohydrin, 2 ml of total reaction mixture). In both cases, pale yellow clear solutions were obtained and their compositions were further analyzed by NMR analysis. The cyclic carbonates were also synthesized using ^13^C isotope enriched CO_2_ (tracer technique) following the experimental procedure that previously used for the similar synthesis with normal or non-^13^C enriched CO_2_ in DMSO. The NMR analysis was carried out with a glass capillary filled with D_2_O for the reactions performed in DMSO solvent or with DMSO-d^6^ where the synthesis was carried out with solution of DBU in alkylene halohydrins.

#### 2.2.2 Separation of EC or PC from the reaction mixture

The separation of EC or PC and recovery of DBU was performed only for the reactions preceded with solution of DBU in alkylene chlorohydrin. After complete conversion of DBU to its salts *i.e.* [DBUH][X] (X = Cl, confirmed by NMR analysis) (hence formation of EC or PC), the reaction mixture added slowly in ethyl acetate where white coloured solid of [DBUH][Cl] got precipitate out. The [DBUH][Cl] was separated by filtration and washed 2 to 3 times with fresh ethyl acetate. The [DBUH][Cl] was further exposed to high vacuum for complete removal of the traces of ethyl acetate. Further, from the collected filtrate, the ethyl acetate was separated from the EC or PC and excess of alkylene chlorohydrin by rotation evaporator. The excess of alkylene chlorohydrin was separated from EC or PC by high vacuum distillation at 40 °C. In order to avoid loss of alkylene chlorohydrin during the distillation, the glass collector was used before vacuum pump where the glass collector was kept in acetone–dry ice mixture. The purity of the obtained products *i.e.* EC or PC as well as remaining [DBUH][Cl] were confirmed by NMR analysis (with DMSO-d^6^ as an internal reference). Further the % recovery of the EC or PC was calculated by the eqn (1).1
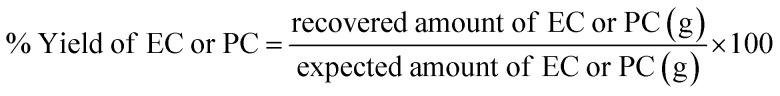


#### 2.2.3 Recovery of DBU

The DBU was recovered from [DBUH][Cl] by using alkaline NaCl saturated aqueous solution following a previously reported procedure.^17^ Initially, saturated NaCl aqueous solution was prepared by mixing 36 g of NaCl in 100 ml of water under stirring. The required amount of NaCl solution was taken to make 4 wt% solution of NaOH in a NaCl saturated solution. The [DBUH][Cl] was added in the alkaline NaCl saturated solution and the mixture was stirred for 30 min at room temperature. The DBU was extracted with ethyl acetate and separated from the aqueous phase by using a separating funnel. The DBU was obtained from organic phase after removing ethyl acetate by rotation evaporator and its purity was confirmed by NMR analysis. The % recovery of the DBU was calculated by the eqn (2).2
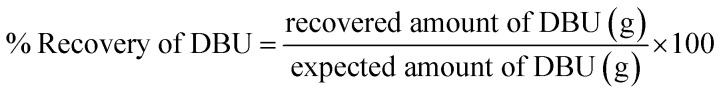


## 3. Result and discussion

The synthesis of cyclic carbonates such as EC or PC was preceded by the introduction of organic superbase DBU through the interaction between molecular CO_2_ and alkylene chlorohydrins such ethylene or propylene chlorohydrins, respectively. Initially, the synthesis of cyclic carbonates was performed in a DMSO solvent where equivalent mixture of DBU and alkylene chlorohydrin were dissolved.

**Scheme 2:**
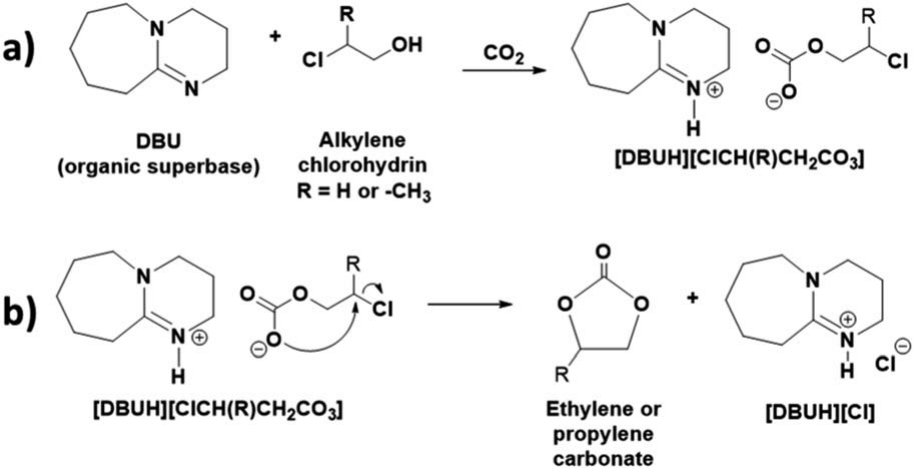
Plausible mechanism for the synthesis of ethylene or propylene carbonate from chlorohydrin and CO_2_. Formation of (a) switchable ionic liquid (SIL), [DBUH][ClCH(R)CH_2_CO_3_] and, (b) ethylene or propylene carbonate.

The primary aim of the use of DBU was to activate the CO_2_ molecule prior to formation of cyclic carbonates. This was facilitated *via* the formation of a switchable ionic liquid (SIL) through equivalent interaction between DBU, alkylene chlorohydrin and CO_2_. The synthesis of organic superbase comprised SIL is a promising approach for the chemical activation of CO_2_ and its storage under mild reaction conditions.^18^ In general, being a superbase, DBU is able to abstract the proton of –OH group of an alcohol (low and high molecular weight alcohols as well as biomolecules such as cellulose) and the thus formed alkoxide anion subsequently interacts with CO_2_ and form SIL *i.e.* [DBUH][alkyl carbonate] (reaction Scheme 2a).^19^

**Fig. 3:**
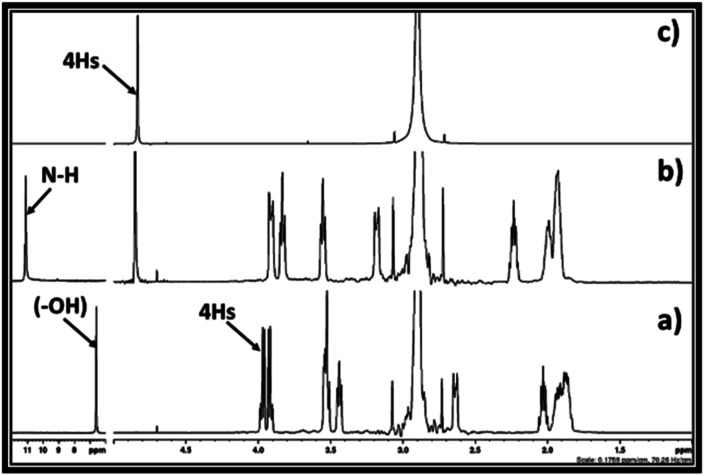
^1^H NMR spectra for (a) equivalent mixture of DBU and ethylene chlorohydrin, (b) reaction mixture after bubbling of CO_2_ in DBU and ethylene chlorohydrin, and (c) commercially available ethylene carbonate in DMSO solvent. (NMR analysis with D_2_O capillary).

In this report, as shown in the reaction Scheme 2, the synthesis of cyclic carbonates from DBU, alkylene chlorohydrin and CO_2_ was initially expected to occur in two steps. The process involves the formation of SIL *i.e.* [DBUH][ClCH(R)CH_2_CO_3_] (R = –H or –CH_3_) at room temperature, followed by the synthesis of cyclic carbonates through release of [DBUH][Cl] under thermal treatment with or without use of catalysts. However, based on the NMR analysis of the reaction mixture, it was observed that during bubbling of CO_2_ in the reaction mixture, the synthesis of cyclic carbonate took place in line with a one-pot process. As shown in Fig. 3a and b, the ^1^H NMR spectra reveals that after interaction of DBU and ethylene chlorohydrin with CO_2_ at room temperature, the two doublets signals of four proton atoms (3.88–4.10 ppm) as well as singlet for the proton in hydroxyl group (6.59 ppm, broad) in ethylene chlorohydrin disappeared. On the other hand, two new signals with the chemical shifts 4.85 (intense) and 11.11 ppm (broad) were observed. Further, after comparing the ^1^H NMR spectra of the reaction mixture with the ^1^H NMR spectra of commercially available EC, the similar intense signal at the 4.85 ppm was also observed for the commercially available EC. The singlet for the 4 proton atoms in the EC appeared as all these protons have identical chemical environment. The signal observed at 11.11 ppm is related to the proton attached to the quaternized and sp^2^-hybridised nitrogen atom in protonated DBU *i.e.* [DBUH]^+^ cation.^20^ The occurrence of the EC and [DBUH]^+^ cation were also further confirmed from the ^13^C NMR analysis.

**Fig. 4:**
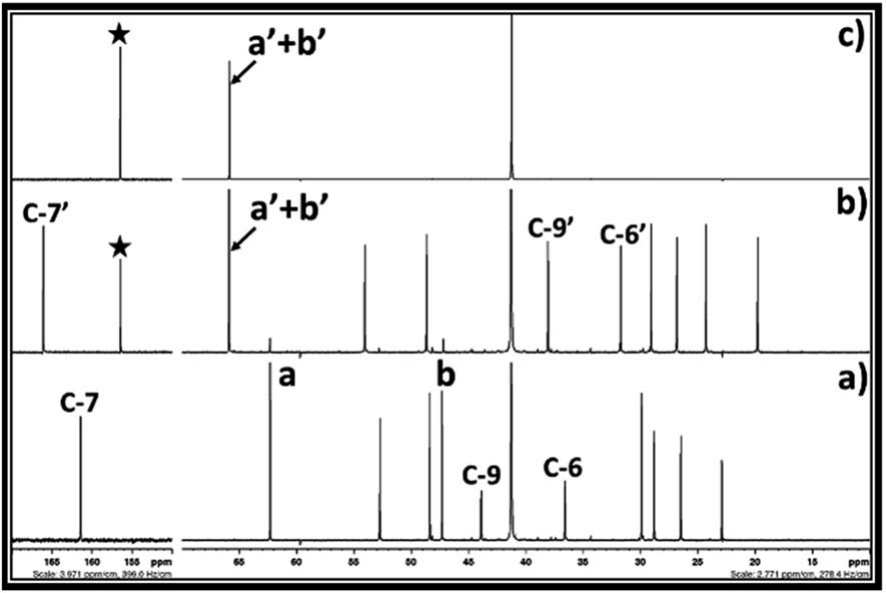
^13^C NMR spectra for (a) equivalent mixture of DBU and ethylene chlorohydrin, (b) reaction mixture after bubbling of CO_2_ in DBU and ethylene chlorohydrin, and (c) commercially available ethylene carbonate in a DMSO solvent. (NMR analysis with D_2_O capillary).

The ^13^C NMR spectra for the reaction mixture obtained after reaction of DBU, ethylene chlorohydrin and CO_2_ in DMSO is shown in Fig. 4. The signals for the non-identical carbon atoms denoted by C-a and C-b in ethylene chlorohydrin were observed at 47.36 and 62.31 ppm, respectively (Fig. 4a and b). After bubbling of CO_2_ in the DMSO solution of the equivalent mixture of DBU and ethylene chlorohydrin, similar to ^1^H NMR spectra, the signals for C-a and C-b disappeared and new signals at 65.94 and 156.40 ppm were observed. After comparison between the NMR spectra of the reaction mixture and commercially available EC, it was observed that the signals appearing at 65.94 and 156.40 ppm, respectively, belong to the aliphatic (C-a′ and C-b′) and carbonyl carbon (denoted by star) of the EC molecule, respectively (Fig. 4b and c). Further, after the interaction of DBU and ethylene chlorohydrin with CO_2_, the carbon atoms in the DBU molecule, at position 6 and 9, got shielded while the carbon atom at position 7 was deshielded which confirmed the formation of [DBUH]^+^ cation.^20^ Hence, based on both ^1^H and ^13^C NMR spectra, the EC was formed after equivalent interaction between DBU, ethylene chlorohydrin and CO_2_ molecule in a one-pot process.

Based on the NMR analysis, even though EC formed in the reaction mixture, it was not confirmed that the synthesis was preceded either through two sub-steps mentioned in the Scheme 2 or other alternative pathways. Therefore, in order to confirm the mechanism of the formation of EC, CO_2_ was bubbled though equivalent mixture of DBU and ethanol (non-chlorinated analogue of ethylene chlorohydrin). As shown in Fig. S1,† although SIL *i.e.* [DBUH][CH_3_CH_2_CO_3_] formed after interaction of equivalent mixture of DBU and ethanol with CO_2_, unlike in the EC synthesis, the isolated signals for the carbon atoms C-a′ and C-b′ in the [CH_3_CH_2_CO_3_]^−^ anion were observed in the ^13^C NMR spectra. Hence, due to absence of leaving group *i.e.* Cl atom at the carbon atom C-a in ethanol, the subsequent intermolecular ring closing for EC synthesis after CO_2_ capture was not observed. However, as [DBUH][CH_3_CH_2_CO_3_] formed in this control experiment and also [DBUH][Cl] formed in the EC synthesis process, we can conclude that the formation of EC proceeds through the two sub-step comprised concerted mechanism shown in reaction Scheme 2. Hence, as shown in the reaction Scheme 2, initially it seems that the SIL forms in the reaction mixture. Further due to presence of Cl atom as a good leaving group, the negatively charged oxygen atom in the carbonate anion induced a nucleophilic attack at the carbon atom to which Cl atom is attached and formation of EC took place through the release of [DBUH][Cl]. Identical mechanism was also proposed previously in case of metal carbonate and amine catalysed processes.^12–15^

**Fig. 5:**
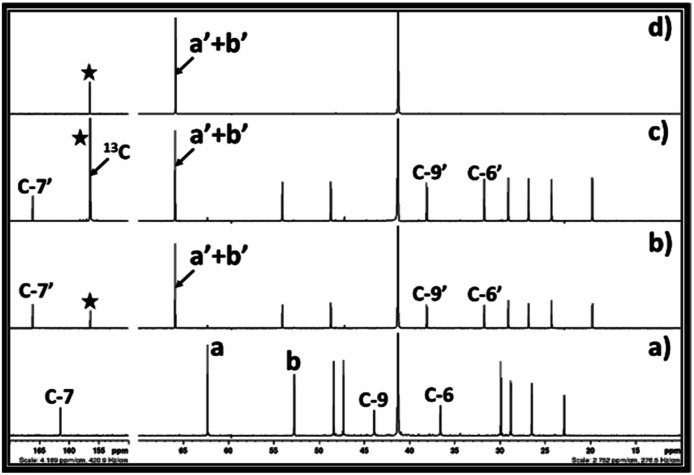
^13^C NMR spectra for (a) equivalent mixture of DBU and ethylene chlorohydrin, reaction mixture after bubbling of (b) CO_2_, (c) ^13^C enriched CO_2_ in DBU and ethylene chlorohydrin, and (d) commercially available ethylene carbonate in DMSO solvent. (NMR analysis with D_2_O in capillary).

Further, in order to confirm the origin of carbon atom in the EC, the ^13^C enriched CO_2_ was used as a CO_2_ source (tracer technique). The ^13^C enriched CO_2_ was bubbled in the equivalent mixture of DBU and ethylene chlorohydrin in DMSO and the one as well as two dimensional NMR analysis of the reaction mixture was carried out. As shown in Fig. 5c, after the use of ^13^C enriched CO_2_, a similar peak pattern was observed for the reaction mixture after comparing it with the NMR of the reaction mixture when ‘normal’ (non-^13^C enriched) CO_2_ was used. However, in case of ^13^C enriched CO_2_, the signal observed for the carbonyl carbon in the EC at the chemical shift 156.40 ppm was having a high intensity compared to the signal for the C-7′ in the [DBUH]^+^ cation (Fig. 5c). On the contrary, in case of the reaction mixture obtained after use of normal CO_2_, the intensity of the signal at 156.40 ppm was found comparable with the intensity of the signal for C-7′ in [DBUH]^+^ cation. In addition, it was also observed that the protons in the EC showed the doublet signal at the chemical shift 4.85 ppm in the ^1^H NMR spectra when ^13^C enriched CO_2_ was used in the synthesis (ESI S2-a†). In this case, the carbonyl carbon atom of EC contained a higher percentage of ^13^C isotope than ^12^C and as the protons of EC are in adjutants to this ^13^C enriched carbonyl carbon atom, these protons showed doublet signal in the NMR spectra.^21^ On the other hand, the signal for these protons was observed as a singlet at the same chemical shift in the ^1^H NMR of the reaction mixture when normal CO_2_ was used (Fig. 2b). So, these observations proved that the EC was formed from the ^13^C enriched CO_2_ and ethylene chlorohydrin through the equivalent interaction with DBU.

The two dimensional correlation NMR analysis techniques such as ^1^H–^1^C HSQC (heteronuclear single quantum coherence) and HMBC (heteronuclear multiple bond correlation) of the reaction mixture was performed when ^13^C enriched CO_2_ was used. This analysis was carried out to get more proof about the formation of EC in the reaction mixture. As shown in Fig. S2-b,† the HSQC NMR analysis represents that the signal for the correlation between four equivalent protons and carbon atoms (C-a′ and C-b′) are observed with the chemical shift at 4.85/65.94 ppm. Further, in case of HMBC NMR analysis, the protons in the EC gave rise to long range coupling with the ^13^C enriched carbonyl carbon atom in the EC and corresponding correlation signal observed at chemical shift 4.85/156.44 ppm (Fig. S2-c†).

Hence, from all these observations based on the both one and two dimensional NMR analysis, it is confirmed that the synthesis of EC proceeds through simultaneous SIL formation followed by intermolecular ring closing reaction shown in the reaction Scheme 2 where chemisorbed CO_2_ became a part of the structure of thus synthesised EC.

**Fig. 6:**
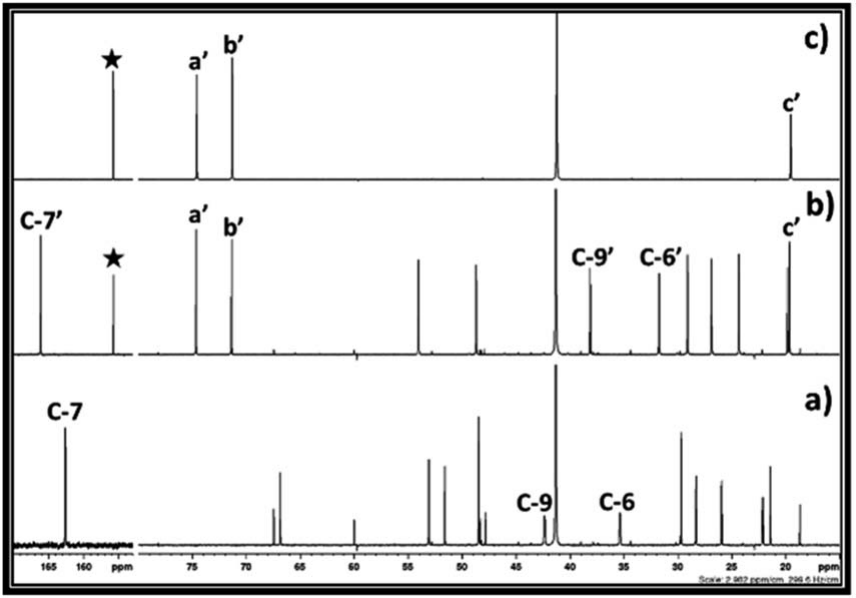
^13^C NMR spectra for (a) equivalent mixture of DBU and propylene chlorohydrin, (b) reaction mixture after bubbling of CO_2_ in DBU and propylene chlorohydrin, and (c) commercially available propylene carbonate in DMSO solvent. (NMR analysis with D_2_O in capillary).

After synthesis of EC from CO_2_ and ethylene chlorohydrin, the similar reaction approach was further mimicked upon synthesis of PC. In this case, propylene chlorohydrin was used as an alkylene chlorohydrin precursor. However, unlike ethylene chlorohydrin, propylene chlorohydrin purchased from Sigma Aldrich comprised of its two different isomers *i.e.* 1-chloro-2-propanol (70 wt%) of and 2-chloro-1-propanol (30 wt%). During actual experiment, it was assumed that the one type of isomer of propylene chlorohydrin was present in the purchased sample container and based on the equivalent of DBU, accordingly the amount of the propylene chlorohydrin was taken for the experiment. As shown in Fig. 6a, the ^13^C NMR spectra also showed that mixed signals for the both types of isomers of propylene chlorohydrin were observed with the signals of DBU. Further CO_2_ was bubbled in the equivalent mixture of DBU and propylene chlorohydrin in DMSO and the formation of PC was confirmed by NMR analysis. The observed ^1^H and ^13^C NMR are shown in the ESI S3† and Fig. 6, respectively. As depicted in Fig. 6a and b, the signals for the both the types of isomers of propylene chlorohydrin disappeared. After comparing the NMR spectra of both the reaction mixture and the commercially available PC sample, it was observed that the PC formed in the reaction mixture (Fig. 6b and c). Also, similar to the EC synthesis, based on the NMR analysis, [DBUH][Cl] also formed in the reaction mixture (Scheme 2 and Fig. 6b). Hence, independent on the type of isomer, it was observed that the PC can be synthesised from mixtures of isomers of propylene chlorohydrin, DBU and CO_2_ following the reaction Scheme 2.

Similar to ^13^C NMR, the ^1^H NMR spectra of the reaction mixture also gave rise to similar observation where prior to CO_2_ exposure, the reaction mixture represented characteristic signal for DBU as well as for both the isomers of propylene chlorohydrin (Fig. S3-a†). After the formation of the PC, the signals belonging to the both isomers of the propylene chlorohydrin disappeared and new deshielded signals for the protons in the PC were observed (Fig. S3-b†). Further, it was also observed that the two protons at the carbon atom C-a in the propylene chlorohydrin became non-identical after formation PC and this was further confirmed with commercially available PC.

**Fig. 7:**
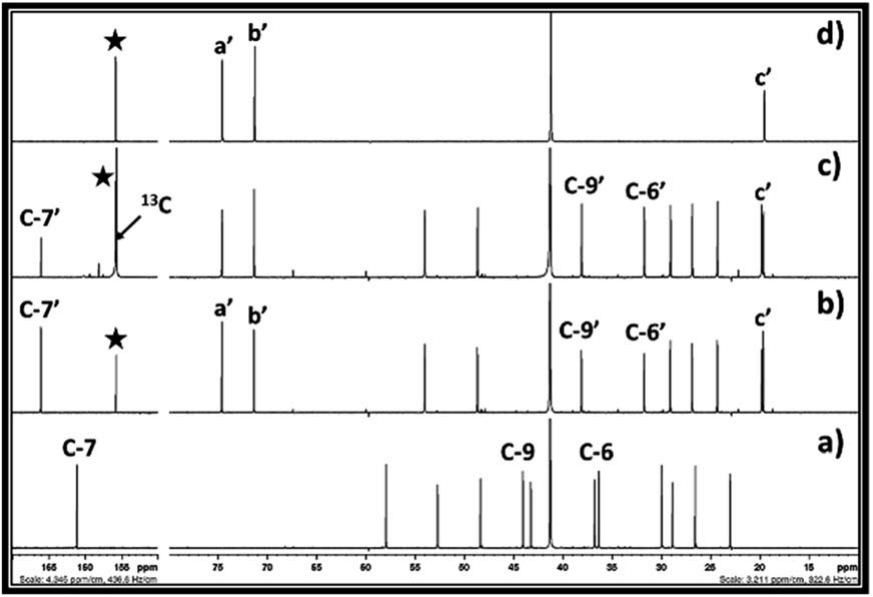
^13^C NMR spectra for (a) equivalent mixture of DBU and propylene chlorohydrin, reaction mixture after bubbling of (b) CO_2_, (c) ^13^C enriched CO_2_ in DBU and propylene chlorohydrin, and (d) commercially available propylene carbonate in DMSO solvent. (NMR analysis with D_2_O in capillary).

Hence, based on the NMR analysis, it was preliminary concluded that PC formed form the equivalent interaction between DBU, propylene chlorohydrin and CO_2_. However, in order to get more confirmation about the origin of the CO_2_, the ^13^C enriched CO_2_ was used in the PC synthesis process and the reaction mixture analysed by one and two dimensional NMR analyses. As shown in Fig. 7c, after the use of ^13^C enriched CO_2_, the intensity of the carbonyl carbon of the PC was found higher than the intensity of the carbon atom C-7′ in the [DBUH]^+^ cation. Meanwhile upon use of normal CO_2_, the intensities of the carbonyl carbon atoms in PC and C-7′ of [DBUH]^+^ cation were found nearly identical to each other (Fig. 6b). Further, similar to EC synthesis, upon use of ^13^C enriched CO_2_ and normal CO_2_, the protons at the positions ‘a’ and ‘b’ in the synthesised PCs showed different splitting patterns in their ^1^H NMR spectra. In case of normal CO_2_, both protons at the position a′ gave rise to triplets while multiplate signal was observed for the proton at position b′ (Fig. S4-a†). On the other hand, upon use of ^13^C enriched CO_2_, the triplet of doublets and broadened singlet were observed for the protons at the positions a′ and b′, respectively (Fig. S4-a†). Hence, the NMR analysis confirmed that the PC was formed from ^13^C enriched CO_2_ and isomers of propylene chlorohydrin through their equivalent reaction with DBU.

Moreover, two-dimensional NMR analysis such as ^1^H–^13^C HSQC and HMBC NMR analysis was also used to highlight better the existence of PC in the reaction mixture. In this case, the two dimensional NMR analysis of the reaction mixture was carried out in which ^13^C enriched CO_2_ was used in the PC synthesis. As depicted in Fig. S4-b,† the HSQC NMR analysis reveals the correlation signals between the proton and carbon atom, at the positions ‘a’ and ‘b’ and gave rise to the chemical shifts 4.42 or 4.95/71.33 (for two different protons at C-a′) and 5.26/74.57 ppm, respectively. The HMBC NMR analysis also confirmed the signals for the long range correlation between the protons atom at the position ‘a’ and ‘b’ and ^13^C enriched carbonyl carbon in PC (Fig. S4-c†).

Hence, similar to EC synthesis, the one and two dimensional NMR analysis confirmed that the PC was formed from the equivalent interaction between DBU, propylene chlorohydrin and CO_2_ where captured CO_2_ remained as a part of PC (reaction Scheme 2).

In order to understand the substrate scope for the DBU based synthesis of cyclic carbonates, various halohydrins such as 2-bromoethanol, 2-iodoethanol, 3-chloro-1-propanol and 3-bromo-1-propanol were also used. The formation of the corresponding cyclic carbonates was confirmed by NMR analysis. In case of both 2-bromo- and 2-iodoethanol, it was observed that after their addition to DMSO solution of DBU, the sp^2^-N atom of the DBU molecule became quaternized (ESI S5 and S6†). This was confirmed by the change in the chemical shifts of carbon atoms of DBU in ^13^C NMR at positions 6, 7 and 9, respectively. Further, after bubbling CO_2_ in the reaction mixture, the peak positions did not change and the peak belonging to the carbonyl carbon atom for the carbonate species was also not observed. Hence, these observations confirmed that the sp^2^-N atom in the DBU molecule become alkylated (*n*-alkylation) through nucleophilic attack on the carbon atom C-a in 2-bromo- or 2-iodoethanol. Therefore, the sp^2^-N in DBU molecule did not remain available to abstract the proton from the hydroxyl group in these halohydrins during the CO_2_ capture process. As Br and I are considered as better leaving groups than Cl, 2-chloroethanol was converted to a cyclic carbonate following the reaction Scheme 2 whereas its bromine and iodine analogues were not able to form cyclic carbonates and converted to *n*-alkylated DBU salts. Hence, it is proven that unlike 2-chloroethanol, 2-bromo 2-iodoethanol cannot be considered as a substrate for the synthesis of cyclic carbonates following the DBU involving synthesis approach.

**Fig. 8:**
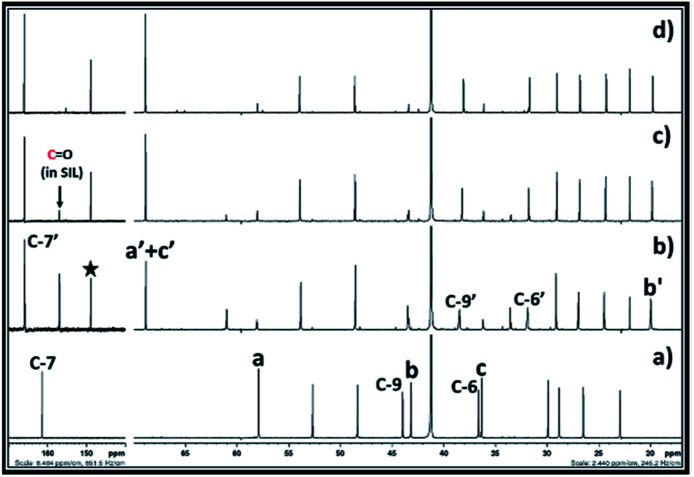
^13^C NMR spectra for (a) equivalent mixture of DBU and 3-chloro-1-propanol in DMSO, reaction mixture of DBU and 3-chloro-1-propanol in DMSO after bubbling of CO_2_ for (b) 20 min, (c) 45 min and, (d) 1 h. (NMR analysis with D_2_O in capillary).

In case of 1,3 halohydrin, as shown in ^13^C NMR (Fig. 8) and ^1^H (ESI S7†), it is possible to synthesise PC from 3-chloro-1-propanol. However, unlike 2-chloroethanol and 1-chloro-2-propanol, the rate of formation of cyclic carbonate *i.e.* PC was slow. From the ^13^C NMR analysis, it was observed that the SIL was formed immediately after the interaction of 3-chloro-1-propanol and DBU with CO_2_. In this case, after the formation of SIL, the peak for the carbonyl carbon atom in the carbonate species was observed with the chemical shift 156.9 ppm. In addition, the peak for the carbon atom C-7 in DBU with the chemical shift 161.4 ppm disappeared while new peak belonging to the carbon atom at position 7 (C-7′) in protonated DBU was observed at 165.8 ppm. Simultaneously, it was also observed that in 20 min PC was formed in the reaction mixture where the peak for the corresponding carbonyl carbon atom was observed at 148.9 ppm (shown by filled star). Further, with an increase in time, the amount of propylene carbonate increased steadily in the reaction mixture as the peak height at chemical shift 148.9 ppm increased. However, even after 1 h of reaction, complete conversion of SIL to cyclic carbonate was not observed. Hence, these observations confirmed that the immediate formation of SIL between DBU, 1-chloro-3-propanol and CO_2_ occurred immediately, but the subsequent cyclisation in the anion of SIL to form propylene carbonate was found as a slow step in this synthesis compared 1,2 chlorohydrins. The reason could be a different position of leaving group *i.e.* Cl atom in the 1-chloro-3-propanol molecule compared other 1, 2 chlorohydrins. Compared to 1,2 chlorohydrins, having one more methylene group at the position ‘b’ in 1-chloro-3-propanol increases the electron density at the carbon atom at position ‘a’ to which Cl atom is attached. This renders the Cl atom as a less good leaving group which reduces further the rate of formation of propylene carbonate compared to the 1,2 chlorohydrins. The use of 3-bromo-1-propanol, on other hand, gave rise to a faster reaction rate than 3-chloro-1-propanol where in 20 minutes it was converted to PC (ESI S8†). In this case, Br is considered as a better leaving group than Cl and, therefore, beyond the structural similarities, the Br containing 1,3 halohydrin showed faster reaction kinetics in the synthesis of PC compared to its Cl containing analogue.

Hence, similar to previously reported metal based reaction approach, organic superbase DBU was also used in the synthesis of cyclic carbonates using various types of halohydrin substrates. In this case, both 1,2 and 1,3 chloro- and bromohydrin except 2-bromo- and 2-iodoethanol were found as useful substrates in the synthesis following the reaction Scheme 1. In case of 1,3 halohydrins, the previous studies involving metal carbonate have controversial results where Cs_2_CO_3_ was able to convert both 1,2 and 1,3 halohydrins to cyclic carbonates. On the other hand, K_2_CO_3_ promoted only reactions with 1,2 halohydrins to corresponding cyclic carbonates. In this study, DBU based reaction approach except in the case of 2-bromo- and 2-iodo ethanol, was able to convert various types of halohydrins to cyclic carbonates and with the faster reaction kinetics compared to metal based processes.

Although the synthesis of both EC and PC was successfully demonstrated following mild reaction approach, the separation of both these cyclic carbonates from DMSO solvent is an energy intensive process. In other word, cyclic carbonates as well as DMSO are considered as high boiling liquids and, therefore, their separation from each other is not feasible through distillation. Also, the separation using solvent extraction can also be a difficult as EC or PC and DMSO are soluble in most of the organic solvents as well as in water. Further, DMSO is not a green solvent, especially in bulk scale synthesis, when its toxic nature as well as expensiveness are considered. Hence, rather than searching alternative techniques for the separation of DMSO, we decided to replace the DMSO with an excess of alkylene chlorohydrin in the reaction mixture. We have also previously used a similar reaction approach upon acrylic plastic precursor synthesis where excess of methanol was used as the solvent to replace DMSO.^22^ Herein we also proposed that the methanol not only performed as a solvent media but also served as a reagent in the process. Considering this, the synthesis of EC or PC was carried out in the alkylene chlorohydrin solvent where the 50 vol% solution of DBU in the ethylene or propylene chlorohydrin was used, respectively.

**Fig. 9:**
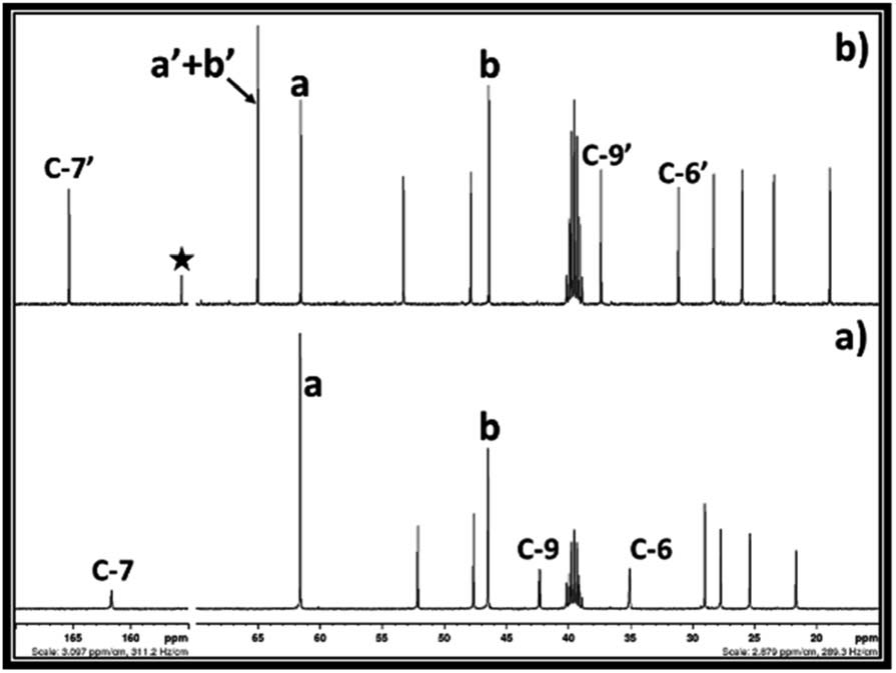
^13^C NMR spectra for (a) 50 vol% solution of DBU in the ethylene chlorohydrin and, (b) reaction mixture after bubbling of CO_2_ in 50 vol% solution of DBU in the ethylene chlorohydrin (NMR analysis in DMSO-d^6^).

**Fig. 10:**
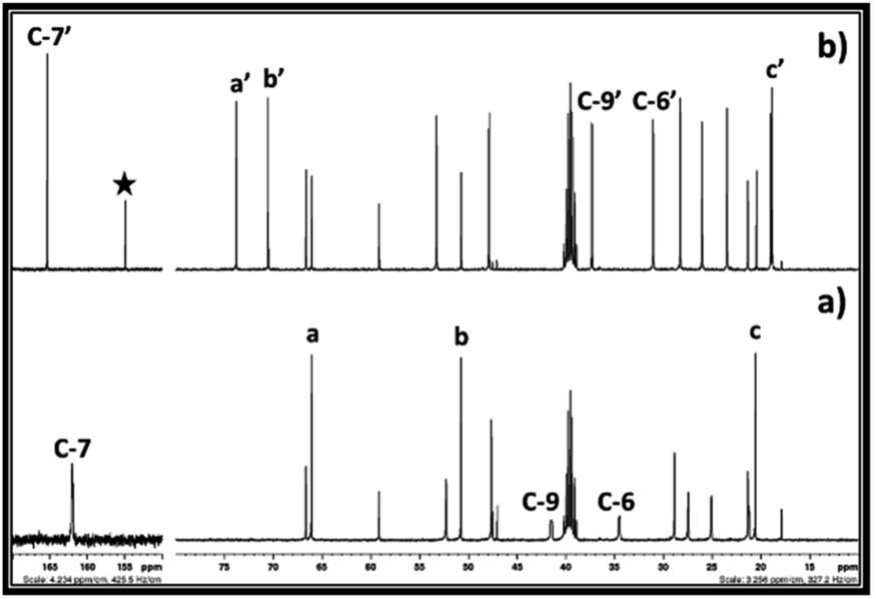
^13^C NMR spectra for (a) 50 vol% solution of DBU in the propylene chlorohydrin and, (b) reaction mixture after bubbling of CO_2_ in 50 vol% solution of DBU in the propylene chlorohydrin (NMR analysis in DMSO-d^6^).

After exposure of the solution of DBU in alkylene chlorohydrin to CO_2_, the formation of EC or PC was confirmed by NMR analysis. The ^13^C and ^1^H NMR spectra for the synthesis of EC in the ethylene chlorohydrin solution are shown in Fig. 9 and in the ESI Fig. S9,† respectively. Similar to synthesis in DMSO, the NMR analysis confirms that EC was formed when the solution of DBU in ethylene chlorohydrin reacted with CO_2_. The ^13^C NMR analysis of the reaction mixture gave rise to isolated signals for both EC and [DBUH][Cl] as well as for the unreacted ethylene chlorohydrin. Similar to synthesis of EC, ^1^H and ^13^C NMR analysis also confirmed the formation of PC after interactions between the solution of DBU in propylene chlorohydrin and CO_2_ (Fig. 10 and S10†). As shown in Fig. 10, the NMR spectra confirm that the signals for the PC, [DBUH][Cl] and unreacted isomers of the propylene chlorohydrin were observed. Hence, it can be concluded that DMSO free synthesis of the EC or PC can proceed where alkylene chlorohydrins can be used as both solvents and reagents in these processes.

Further, as discussed previously, excess of alkylene chlorohydrins were used in order to facilitate the separation of components in the reaction mixture after the formation of cyclic carbonates. The separation of the EC or PC, [DBUH][Cl] and the excess of the alkylene chlorohydrins was achieved following the separation approaches mentioned in the experimental section. The composition of the [DBUH][Cl] obtained through its precipitation after addition of ethyl acetate in the reaction mixture was confirmed by ^1^H NMR analysis. As shown in the ESI S12-a,† pure form of [DBUH][Cl] was recovered from the reaction mixture after the synthesis of the cyclic carbonates. After the removal of the [DBUH][Cl], the remaining ethyl acetate solution was further processed for the recovery of the excess of alkylene chlorohydrin and EC or PC. The ethyl acetate was removed using rotation evaporator followed by alkylene chlorohydrins were separated from the EC or PC using high vacuum distillation process at 40 °C. The composition of the recovered EC or PC were analysed by the ^1^H NMR analysis (Fig. S11†) while their recovery was calculated by eqn (1) (Experimental section). The ^1^H NMR spectra of the EC and PC showed that pure cyclic carbonates were recovered where recovery levels of 92 and 96%, respectively, were reached. The high recovery as well as purity of these cyclic carbonates was achieved because of their high boiling points compared to respective alkylene chlorohydrins. Therefore, high vacuum distillation at 40 °C allowed efficient separation of alkylene chlorohydrins from cyclic carbonates. Hence, use of alkylene chlorohydrin solution of the DBU instead of DMSO in the cyclic carbonates synthesis facilitated the separation of the reaction fractions such as cyclic carbonates and [DBUH][Cl] from each other.

DBU was recovered from the [DBUH][Cl] applying alkaline solvent treatment and solvent extraction process. The purity and the recovery of the DBU was determined by the ^1^H NMR analysis and the eqn (2) (Experimental section). The NMR analysis confirmed that pure DBU was recovered while 76% recovery was achieved (Fig. S12-b†). However, as mentioned in a previous report, the recovery of the DBU using alkaline solvent treatment from its cationic form is inconsistent and not totally optimized.^17^ However, considering the price as well as the importance of DBU in the activation of CO_2_ under mild reaction conditions, more work needs to be performed in order to reach higher recovery of the DBU from [DBUH][Cl].

Hence, in this report, compared to previous study where metal carbonates were used as an activating agent for cyclic carbonate synthesis from halohydrins, the similar synthesis was performed at room temperature and atmospheric pressure with faster reaction kinetics and no use of the metal based catalysts or co-catalysts. As it was described previously, even though metal based synthesis approach for cyclic carbonate synthesis can be applied for various types of halohydrin substrates, it is also accompanied with the generation of the 2 equivalents of metal salts as a waste per equivalent of cyclic carbonate (MHCO_3_ and MCl or MBr where M = K, Na or Cs). In this case, authors also did not describe the recovery of these salts as well as regeneration of active metal carbonate catalysts. Unlike these metal based processes, the generation of waste did not occur in DBU based reaction approach except the formation of easily recoverable by-product such as [DBUH][Cl] salt. Besides that, the recovered DBU salt can be converted to DBU with alkaline solvent treatment. Hence, the route proposed in this report for the synthesis of cyclic carbonates from various chlorohydrins can be more preferable compared to the metal based approach.

## 4. Conclusions

A one-pot and metal free synthesis of the both ethylene carbonate (EC) and propylene carbonates (EC) from molecular CO_2_ was successfully carried out under mild reaction conditions. Various Cl, Br and I comprised 1,2 and 1,3 alkylene halohydrins and organic superbase DBU reacted with CO_2_ to form cyclic carbonate and halide salts of DBU. The NMR based mechanistic studies confirmed that the switchable ionic liquid (SIL) was formed *in situ* from DBU, alkylene halohydrin and CO_2_ and was further converted to EC or PC through intermolecular ring closing reaction. In the case of 1,2 halohydrins, 2-chloroethanol and 1-chloro-2-propanol were converted to EC and PC, respectively, whereas 2-bromo and 2-iodoethanol reacted equivalently with DBU and formed *n*-alkylated of DBU salts instead of cyclic carbonates. Both 1,3 halohydrins such as 3-chloro-1-propanol and 3-bromo-1-propanol could be converted to PC. However due to having Br as a good leaving species compared to Cl, 3-bromo-1-propanol gave rise to faster reaction kinetics compared to 3-chloro-1-propanol during the formation of PC. In order to avoid the use of DMSO as a solvent, the EC or PC synthesis from 1,2 chlorohydrins was further carried out using chlorohydrins as a solvent. In this case, the chlorohydrins not only performed as a solvent media but also served as reagents in the process. Further, the use of chlorohydrins as a solvent also enabled efficient separation of the reaction components through mild experimental approaches. In this process, pure forms of EC or PC were obtained where 92 and 96% of recovery of cyclic carbonates was achieved, respectively. The organic superbase DBU was also recovered with high purity from [DBUH][Cl] using alkaline solvent treatment whereupon the recovery was 76%. Hence, in this report DBU superbase involved synthesis of EC and PC from CO_2_ was carried out using various types of halohydrins. The synthesis was further successfully upgraded using halohydrins as both solvent media and reagent. Being metal free, one pot and mild reaction condition approach as well as having control over the waste generation, this DBU involved reaction approach can be applied in large-scale synthesis of cyclic carbonates from CO_2_ and halohydrins.

## Conflicts of interest

There are no conflicts to declare.

## Supplementary Material

RA-009-C9RA06765E-s001
