# Supplementary material for: Metal free synthesis of ethylene and propylene carbonate from alkylene halohydrin and CO2 at room temperature
Source: RSC Adv. 2019 Oct 22;9(58):34023–31. doi: 10.1039/c9ra06765e (PMC9082319; doi:10.1039/c9ra06765e)

Electronic Supplementary Material (ESI) for RSC Advances.
This journal is © The Royal Society of Chemistry 2019

Supporting information

Title: Metal free synthesis of ethylene and propylene carbonate from alkylene halohydrin and CO_2 at room temperature

Santosh Govind Khokarale, Jyri-Pekka Mikkola

Figure S1- ^{13}C NMR spectra for a) reaction mixture of DBU and ethanol, and b) reaction mixture after bubbling of CO_2 in DBU and ethanol (in DMSO).

Figure S2- a) ^{1}H NMR spectra for reaction mixture after bubbling of CO_2 or ^{13}C enriched CO_2 in DBU and ethylene chlorohydrin (in between 4.4-5.5 ppm), two dimenational NMR analysis of the reaction mixture with ^{13}C enriched CO_2 b) ^{1}H-^{13}C HSQC and, c) ^{1}H-^{13}C HMBC NMR analysis (NMR analysis with D_2O in capillary).

Figure S3- ^{1}H NMR spectra for a) reaction mixture of DBU and propylene chlorohydrin, and b) reaction mixture after bubbling of CO_2 in DBU and propylene chlorohydrin and, c) commercially available propylene carbonate.

Figure S4- a) ^{1}H NMR spectra for reaction mixture after bubbling of CO_2 or ^{13}C enriched CO_2 in DBU and propylene chlorohydrin (in between 4.6-5.0 ppm), two dimenational NMR analysis of the reaction mixture with ^{13}C enriched CO_2 b) ^{1}H-^{13}C HSQC and, c) ^{1}H-^{13}C HMBC NMR analysis (NMR analysis with D_2O in capillary).

Figure S5- ^{13}C NMR spectra of a) DBU, equivalent mixture of DBU and ethylene bromohydrin or 2-iodoethanol b) before and, c) after bubbling CO_2 (NMR analysis with D_2O in capillary).

Figure S6- ^{13}C NMR spectra of a) DBU, equivalent mixture of DBU and ethylene Iodohydrin or 2-iodoethanol b) before and, c) after bubbling CO_2 (NMR analysis with D_2O in capillary).

Figure S7- ^{1}H NMR spectra for a) equivalent mixture of DBU and 3-chloro-1-propanol in DMSO, reaction mixture of DBU and 3-chloro-1-propanol in DMSO after bubbling of CO_2 for b) 20 min, c) 1h and, d) 2h. (NMR analysis with D_2O in capillary)

Figure S8- ^{13}C NMR spectra of equivalent mixture of DBU and 3-bromo-1-propanol in DMSO a) before and, b) after bubbling CO_2 (NMR analysis with D_2O in capillary).

Figure S9- ^{1}H NMR spectra for a) 50 vol.% solution of DBU in the ethylene chlorohydrin and, b) reaction mixture after bubbling of CO_2 in 50 vol.% solution of DBU in the ethylene chlorohydrin.

Figure S10- ^{1}H NMR spectra for a) 50 vol.% solution of DBU in the propylene chlorohydrin and, b) reaction mixture after bubbling of CO_2 in 50 vol.% solution of DBU in the propylene chlorohydrin.

Figure S11- ^{1}H NMR spectra for a recovered a) Ethylene carbonate and, b) Propylene carbonate.

Figure S12- ^{1}H NMR spectra for a recovered a) [DBUH][Cl] and, b) DBU**.**

Figure S1.

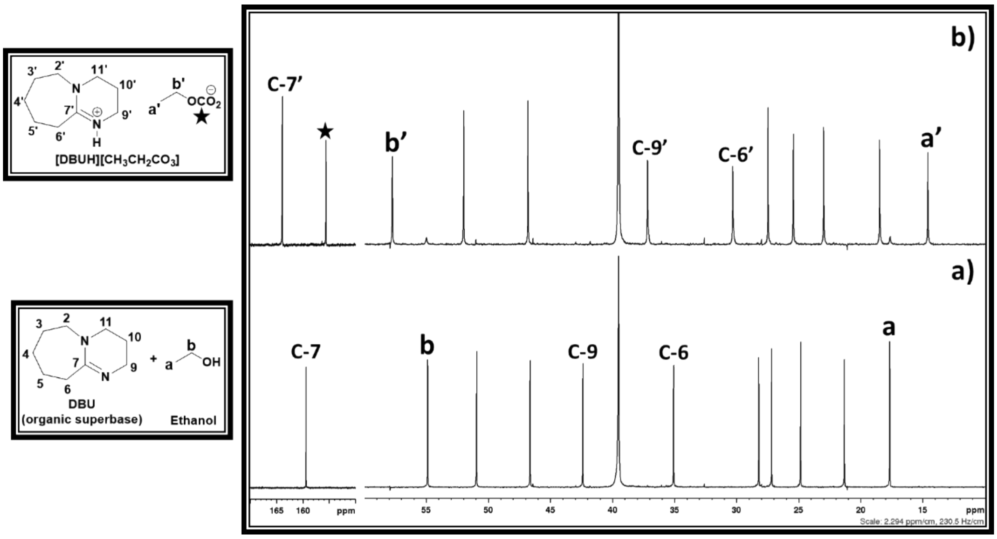

Figure S2:

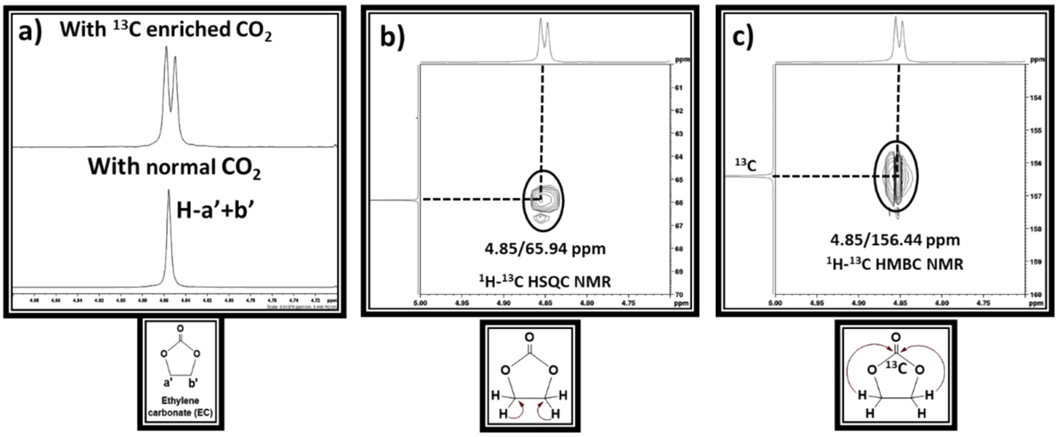

Figure S3.

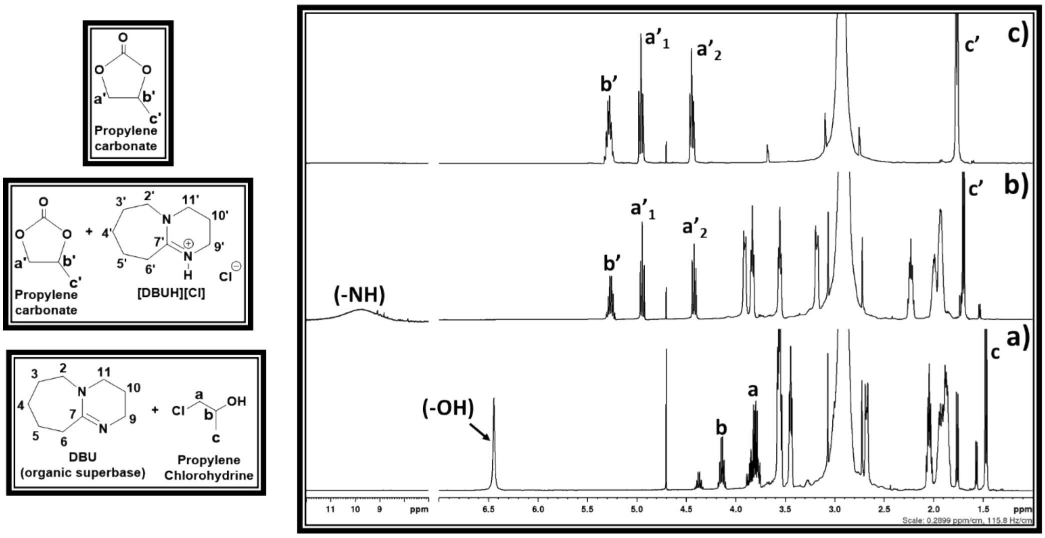

Figure S4:

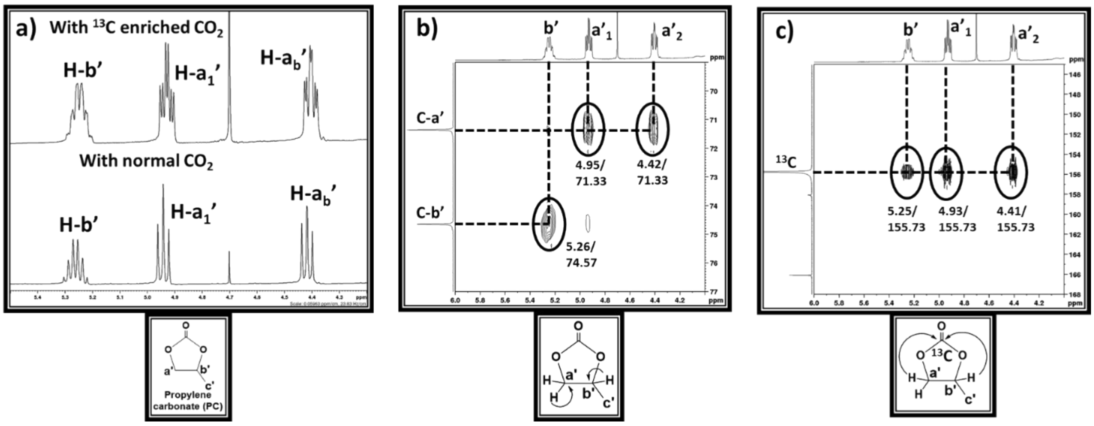

Figure S5

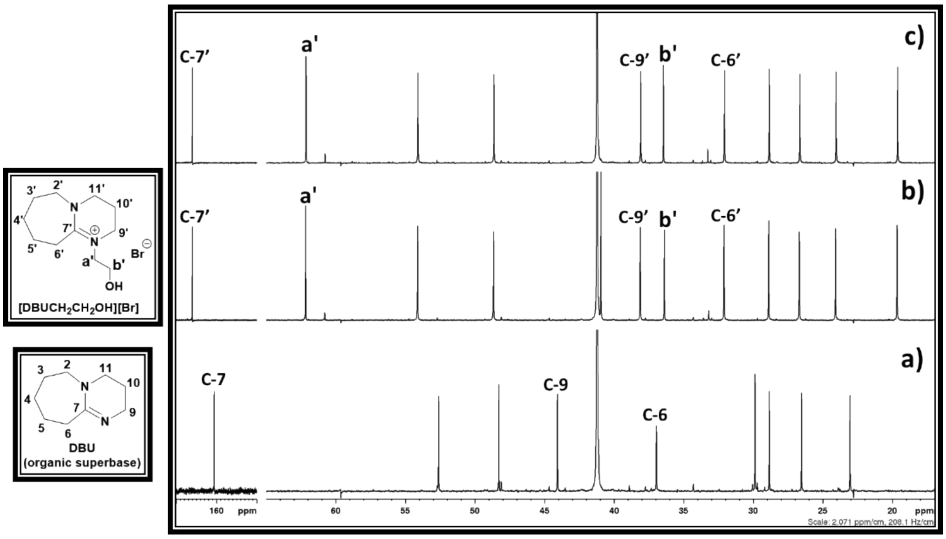

Figure S6

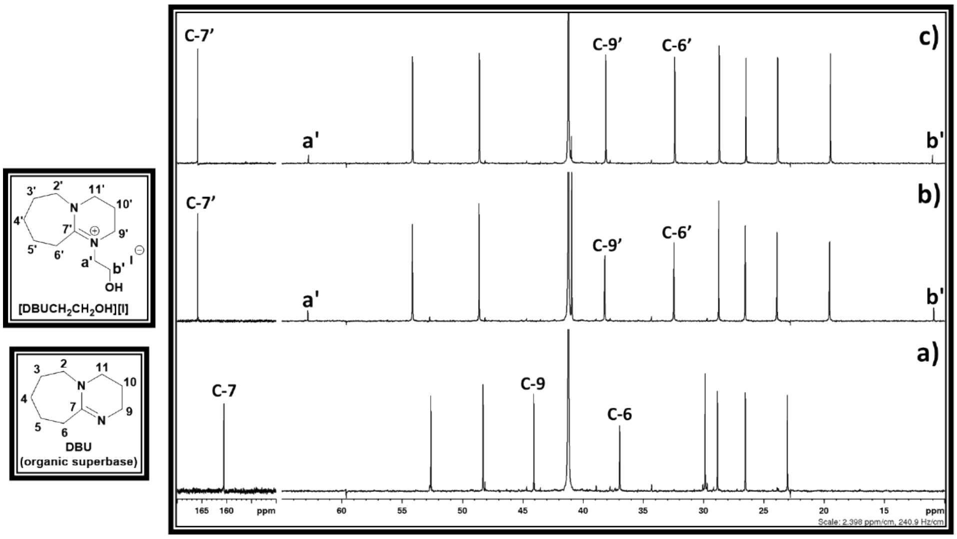

Figure S7

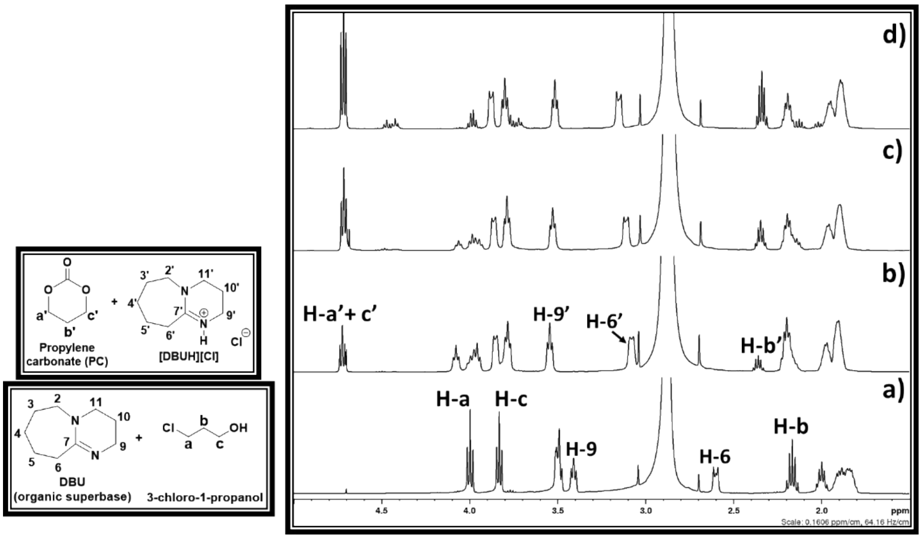

Figure S8

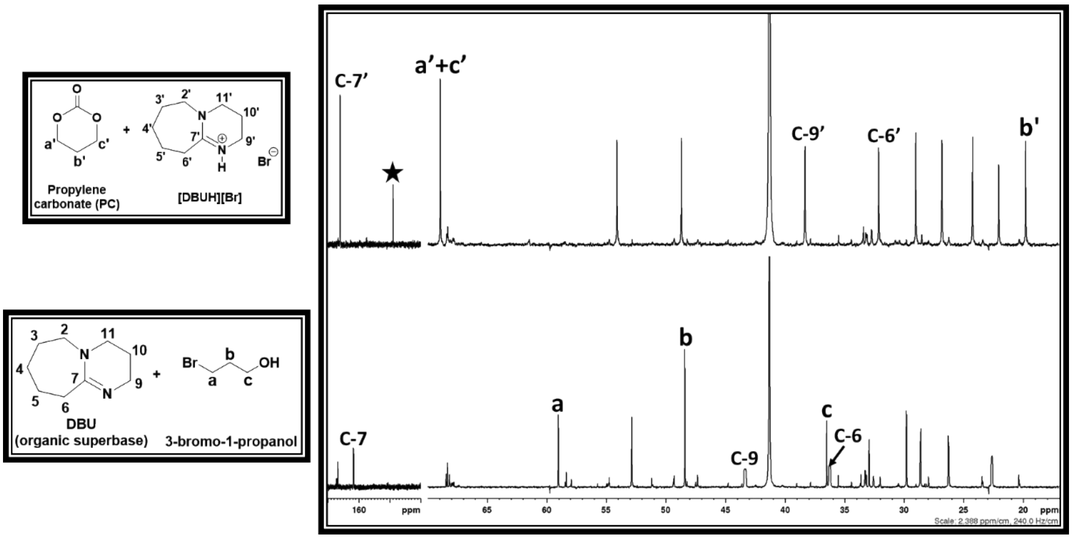

Figure S9.

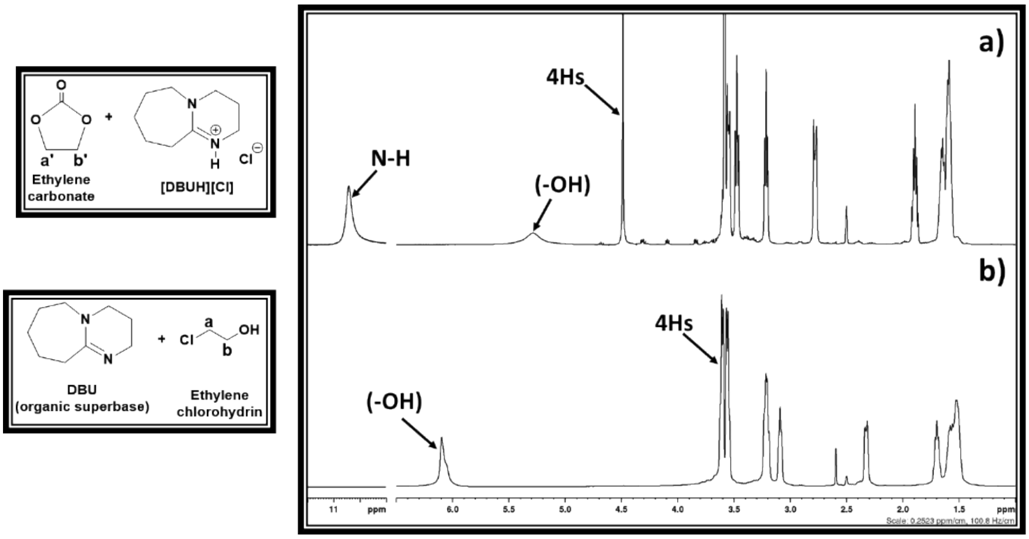

Figure S10.

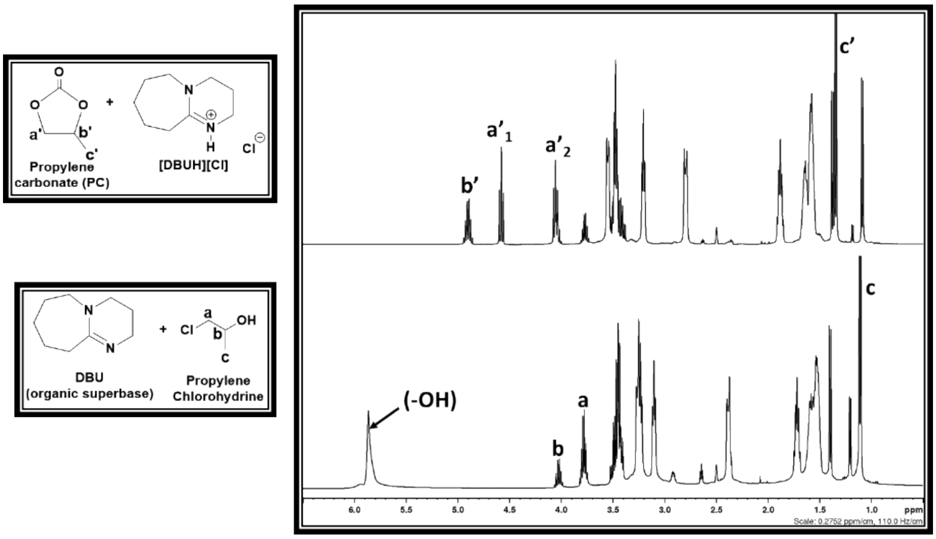

Figure S11.

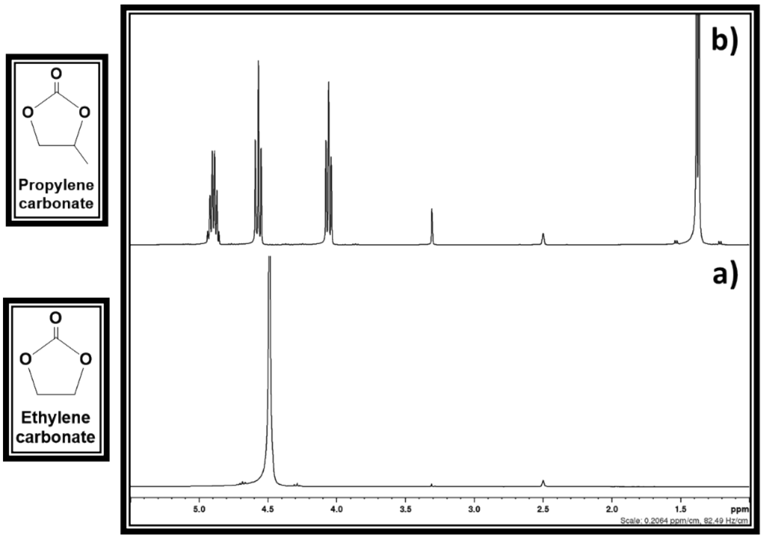

Figure S12.

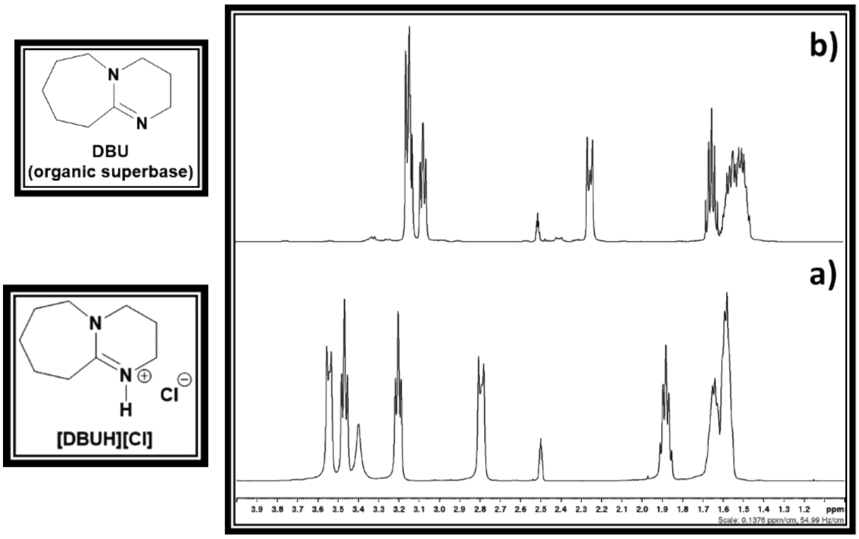

Supplement: RA-009-C9RA06765E-s001 [file RA-009-C9RA06765E-s001.pdf]
